# Telehealth system (e-CUIDATE) to improve quality of life in breast cancer survivors: rationale and study protocol for a randomized clinical trial

**DOI:** 10.1186/1745-6215-14-187

**Published:** 2013-06-22

**Authors:** Noelia Galiano-Castillo, Angelica Ariza-García, Irene Cantarero-Villanueva, Carolina Fernández-Lao, Lourdes Díaz-Rodríguez, Marta Legerén-Alvarez, Carmen Sánchez-Salado, Rosario Del-Moral-Avila, Manuel Arroyo-Morales

**Affiliations:** 1 Department Physical Therapy, University of Granada, Granada, Spain; 2 Physical Medicine and Rehabilitation Department, Clinico Universitario San Cecilio Hospital, Andalusian Health Service, Granada, Spain; 3 Nursing Department, University of Granada, Granada, Spain; 4 Medical Oncology Unit, Clinico Universitario San Cecilio Hospital, Andalusian Health Service, Granada, Spain; 5 Breast Oncology Unit, Hospital Virgen de las Nieves, Granada, Spain; 6 Radiotherapy Oncology Unit, Hospital Virgen de las Nieves, Granada, Spain

**Keywords:** Breast, Neoplasm, Telerehabilitation, Exercise

## Abstract

**Background:**

Breast cancer survivors suffer physical impairment after oncology treatment. This impairment reduces quality of life (QoL) and increase the prevalence of handicaps associated to unhealthy lifestyle (for example, decreased aerobic capacity and strength, weight gain, and fatigue). Recent work has shown that exercise adapted to individual characteristics of patients is related to improved overall and disease-free survival. Nowadays, technological support using telerehabilitation systems is a promising strategy with great advantage of a quick and efficient contact with the health professional. It is not known the role of telerehabilitation through therapeutic exercise as a support tool to implement an active lifestyle which has been shown as an effective resource to improve fitness and reduce musculoskeletal disorders of these women.

**Methods / Design:**

This study will use a two-arm, assessor blinded, parallel randomized controlled trial design. People will be eligible if: their diagnosis is of stages I, II, or IIIA breast cancer; they are without chronic disease or orthopedic issues that would interfere with ability to participate in a physical activity program; they had access to the Internet and basic knowledge of computer use or living with a relative who has this knowledge; they had completed adjuvant therapy except for hormone therapy and not have a history of cancer recurrence; and they have an interest in improving lifestyle. Participants will be randomized into e-CUIDATE or usual care groups. E-CUIDATE give participants access to a range of contents: planning exercise arranged in series with breathing exercises, mobility, strength, and stretching. All of these exercises will be assigned to women in the telerehabilitation group according to perceived needs. The control group will be asked to maintain their usual routine. Study endpoints will be assessed after 8 weeks (immediate effects) and after 6 months. The primary outcome will be QoL measured by The European Organization for Research and Treatment of Cancer Quality of Life Questionnaire Core 30 version 3.0 and breast module called The European Organization for Research and Treatment of Cancer Breast Cancer-Specific Quality of Life questionnaire. The secondary outcomes: pain (algometry, Visual Analogue Scale, Brief Pain Inventory short form); body composition; physical measurement (abdominal test, handgrip strength, back muscle strength, and multiple sit-to-stand test); cardiorespiratory fitness (International Fitness Scale, 6-minute walk test, International Physical Activity Questionnaire-Short Form); fatigue (Piper Fatigue Scale and Borg Fatigue Scale); anxiety and depression (Hospital Anxiety and Depression Scale); cognitive function (Trail Making Test and Auditory Consonant Trigram); accelerometry; lymphedema; and anthropometric perimeters.

**Discussion:**

This study investigates the feasibility and effectiveness of a telerehabilitation system during adjuvant treatment of patients with breast cancer. If this treatment option is effective, telehealth systems could offer a choice of supportive care to cancer patients during the survivorship phase.

**Trial registration:**

ClinicalTrials.gov Identifier: NCT01801527

## Background

Approximately 4.4 million women worldwide live with a diagnosis of breast cancer [1]. Developments in screening and improved treatments for breast cancer have led to improved survival [2,3]. Cancer is increasingly viewed as a chronic disease and therefore there is a growing need for long-term treatments [4,5]. Breast cancer survivors suffer physical impairment after oncology treatment [6,7]. This impairment reduces quality of life (QoL) and increases the prevalence of conditions associated with an unhealthy lifestyle (for example, decreased aerobic capacity and strength, weight gain, and fatigue) [8]. The reduction of range of movement in the neck/shoulder complex and chronic pain in the neck/shoulder complex are the most prevalent musculoskeletal dysfunction associated with oncology treatment [9,10].

There is evidence that physical activity is effective in the primary and secondary prevention of chronic diseases [11]. There is increasing evidence that patients with cancers such as breast, colon, prostate cancer, and hematological malignancies may benefit from physical exercise programs in terms of improvement in fitness levels, physical activity, and health-related quality of life (HRQoL) [4]. In fact, many previous studies, such as observational studies [12] and randomized controlled trials [13], have assessed the effects of physical activity on treatment-related symptoms and recovery outcomes. For this reason, physical activity has become an aim of cancer recovery research for breast cancer patients [14].

One of common postoperative complications is reduced range of motion (ROM) in the shoulders of breast cancer patients [15-17]. Much research has supported performing early exercises to avoid limitations of ROM in the shoulder [15,18]. This and other impairments may improve with physical activity in women with breast cancer [19] therefore adequate measurements should be implemented to improve disability in breast cancer women.

Recent work has shown that exercise programs after diagnosis of breast cancer adapted to individual characteristics of patients are related to improved overall and disease-free survival [1]. The benefits of regular physical activity as a way of recovery show enough scientific evidence to justify the development of new health services that respond to the needs of these patients after cancer process. However, the implementation of this type of program involves high cost resources that usually are not viable and require that participants live near the facilities which is not possible in non-urban women or women of low socioeconomic status [20].

Nowadays, technological support is a promising strategy that could improve issues such as barriers of distance, time, and cost [21]. Patients have the great advantage of quick and efficient contact with the health professional. On the other hand, the health professional can provide therapeutic intervention more efficiently in response to a patient’s needs. The monitoring of some variables (for example, weight, heart rate, and arm mobility) allows the control of these patients, which may provide improved adhesion of programs that seek to increase the QoL of these patients. The motivation of patients can be increased significantly using the telehealth system by immediate feedback that may be responsible for an active approach to fitness.

A systematic review [22] supports the view that telerehabilitation can lead to similar clinical outcomes compared to traditional rehabilitation programs, with possible positive impact on some areas of healthcare utilization. There are internet-based programs that increase amount of physical activity in different types of diseases such as multiple sclerosis [23] and juvenile idiopathic arthritis [11]. In particular, recent research [24] reported a home-based exercise program that has shown to effectively improve affected upper-limb symptoms (for example, lymphedema) and led to improved QoL of breast cancer patients. The role of telerehabilitation through therapeutic exercise is not known as a support tool to implement an active lifestyle which has been shown as an effective resource to improve fitness and reduce musculoskeletal disorders in these women [25].

It is relevant to the future of patients in a clinical setting to know whether a telerehabilitation intervention to improve physical status and reduce pain shows benefits compared to usual care in breast cancer survivors.

## Methods

### Objectives

The overall objective of the e-CUIDATE telerehabilitation randomized controlled trial is to evaluate the immediate and long-term effects of a telerehabilitation program on the overall impact of QoL, pain, body composition, physical measurement, cardiorespiratory fitness, fatigue, anxiety and depression, cognitive function, and lymphedema. The intervention group will receive three training sessions each week for an 8-week period. We will also study the effect of a 24-week period without telehealth support on the studied variables.We hypothesize that a strategy for care based on telerehabilitation to promote therapeutic exercise will increase QoL, reduce musculoskeletal disorders, and improve fitness in breast cancer patients.

### Research design and methods

A randomized controlled trial will be conducted with assessments at baseline, 4 weeks and immediately after intervention (at 8 weeks). Follow-up measurements will be carried out 24 weeks after the end of the 8-week intervention period, giving a total trial data collection period of 32 weeks. The assessments will be performed on 2 separate days to avoid fatigue in the patients. The study assessment schedule is shown in Table 1.

**Table 1:** Study assessment schedule

**Assessment**	**Baseline**	**Follow-up**	**Post-intervention**	**Detraining**
		**(4 weeks)**	**(8 weeks)**	**(24 weeks)**
Informed consent	X			
*Day 1 testing*				
Sociodemographic data	X			
Anthropometric data, pressure/rate heart	X		X	X
Training on the treadmill	X			
Lymphedema	X		X	X
*Day 2 testing*				
Piper Fatigue Scale-revised	X	X	X	X
Trail Making Test	X		X	X
Auditory Consonant Trigram	X		X	X
EORTC QLQ-C30	X	X	X	X
EORTC QLQ-BR23	X	X	X	X
International Fitness Scale	X		X	X
Brief Pain Inventory short form	X	X	X	X
Hospital Anxiety and Depression Scale	X		X	X
Visual Analogue Scale for pain	X		X	X
Algometry	X		X	X
Body composition	X		X	X
Handgrip strength	X		X	X
Back muscle strength	X		X	X
Multiple sit-to-stand test	X		X	X
Abdominal test	X		X	X
6-min walk test	X		X	X
Borg Fatigue Scale	X		X	X
Accelerometry (1 week)	X		X	X
*Questionnaire to be completed at home after accelerometry*				
Questionnaire	X		X	X
IPAQ-SF Satisfaction survey			X	

### Specific research aims

1. To investigate the effects of telerehabilitation-based controlled exercise and adapt the needs of this population on physical fitness and pain perceived parameters.

2. To assess characteristics of breast cancer survivors more adequate to receive benefits from interactive program of telerehabilitation.

### Participants

A total of 80 breast cancer survivors will be randomized to receive the interactive rehabilitation or usual care. The telerehabilitation group will receive an 8-week interactive intervention and the control group will receive printed material at the beginning of the study. Breast cancer survivors will be recruited through the services of the oncology and breast unit at the Virgen de las Nieves Hospital and San Cecilio Hospital in the province of Granada. This study has been approved by the Medical Ethics Committee of the University of Granada and the local Ethical Boards of the participating hospitals.

### Eligibility criteria

To be eligible for this study participants will need to meet the following criteria: diagnosis of stage I, II, or IIIA breast cancer; medical clearance of participation; free of chronic disease or orthopedic issues that would interfere with ability to participate in a physical activity program; access to the Internet; basic ability to use a computer or living with someone who has this ability; completion of adjuvant therapy except for hormone therapy; no history of cancer recurrence; have interest in improving lifestyle: fitness/stress level; have signed informed consent.

### Assessment

#### Primary outcome measure

##### Quality of life

*The European Organization for Research and Treatment of Cancer Quality of Life Questionnaire Core 30* (EORTC QLQ-C30) *version 3*.*0*[26]: QoL will be assessed using EORTC QLQ-C30. This is one of the most widely-used instruments for measuring QoL in cancer patients. The questionnaire includes both multi-item scales and single-item measures. These are composed by five functional scales, three symptom scales, a global health status/QoL scale, and six single items; the scores have to be averaged and transformed linearly to obtain a range of score from 0 to 100 with higher score meaning a great response level [27].

*The European Organization for Research and Treatment of Cancer Breast Cancer*-*Specific Quality of Life questionnaire* (EORTC QLQ-BR23) [26]: It is a breast cancer module of EORTC QLQ-C30. This module contains 23 items assessing disease symptoms, side effects of treatment, body image, sexual functioning, and future perspective. All items are rated on a four-point scale ranging from 1 (not at all) to 4 (very much). The scoring procedure of the breast cancer module is the same as the EORTC QLQ-C30 [27]. The reliability has been found high to moderate (Cronbach´s α ranged between 0.46-0.94) [28].

### Other outcome measures

#### Pain

Algometry is used to measure pressure pain threshold (PPT) [29] levels through an electronic algometer (Somedic AB, Farsta, Sweden). We assess bilaterally PPTs over the C5-C6 zygapophyseal joint, deltoid muscle, and tibialis anterior muscle. The order of assessment for the different points was randomized between participants. The pressure is at an approximate rate applied of 30 kPa/s by a 1 cm^2^ probe. We ask for participants to press the switch when they first feel a change from pressure to pain. The mean of three tests (intra-examiner reliability) is used for the main analysis. There is an interval delay of 30 s between each test [30]. The reliability of pressure algometry has been found to be high the same day (intraclass correlation coefficient=0.91 (95% confidence interval (CI) 0.82-0.97)) [31] and between 4 separate days (intraclass correlation coefficient=0.94-0.97) [32].

The Visual Analogue Scale (VAS) is a scale for subjective pain estimation that consists of scores in the range of 0 to 10 where 0 indicates ‘no pain’ and 10 indicates ‘worst pain imaginable’. Participants mark the level of pain that they feel in that moment for both arms. The VAS has been widely used and has shown to be a reliable and valid instrument for assessing pain.

The Brief Pain Inventory (BPI) short form [33-35]: This version of the BPI has the front and back body diagrams, four pain severity items (assessment the pain in its ‘worst’, ‘least’, ‘average’, and ‘now’) and seven pain interference items with daily activities whose scores range from 0 (no interference) to 10 (interferences completely). Pain severity and pain interference are obtained from mean scores. Furthermore the pain is answered in the last 24 h in this short form and there is an item about percentage of pain relief by treatment.

#### Body composition

Height will be measured. Weight, body mass index, skeletal muscle mass, and percentage of body fat will be obtained with bioelectrical impedance analysis (InBody 720; Biospace, Seoul, South Korea).

#### Physical measurement

The Abdominal test [36]: The subject lie down supine on a bench with knees flexed and heels about 0.30 m from buttocks. The arms have to be lifted with guided palms to level the knees so that the inferior angle of the scapula is barely lifted from the bench. The number of seconds that the position is maintained is recorded.

Measurement of upper body muscular strength: Handgrip strength is determined using digital dynamometer (TKK 5101 Grip-D; Takey, Tokyo, Japan). The precision will be 0.1 kg. For dynamometry measurement, patients maintain the standard bipedal position during the entire test with the arm in complete extension and they do not touch any part of the body with the dynamometer except the hand being measured. The determination of optimal grip span according to hand size is obtained through a simple algorithm which allowing the grip span to adapt to the hand size in women before the test [37]. Each subject will do three tests for each hand (alternating both hands) with 1 min of delay between measures. The final result will be the mean score for each hand. This test is valid and reliable [38].

Measurement of back muscle strength: Back muscle strength is assessed with a digital dynamometer (TKK 5002 Back-A; Takey, Tokyo, Japan). The precision will be 1 kg. The patients have to maintain a standing posture with 30° lumbar flexion [39]. Each subject will do three tests with 1 min of delay between measures. The mean from three tests is recorded.

Lower body endurance: Multiple sit-to-stand test is used to assess general lower extremity endurance [40]. Participants are asked, while sitting at the front of the chair, to rise until they reach full knee extension and sit back 10 times as fast as possible. The length of time taken to complete this will be recorded.This test has shown to be reliable [41].

### Cardiorespiratory fitness

The International Fitness Scale (IFIS) [42,43]: The IFIS is scored on a 5-point Likert scale with five response possibilities (‘very poor’, ‘poor’, ‘average’, ‘good’, and ‘very good’) and deals with perceived patients’ overall fitness, cardio-respiratory fitness, muscular fitness, speed-agility, and flexibility.

Functional capacity: The 6-min walk test using a treadmill (H-P-COSMOS for graphics; Germany) consists of determining the maximum distance (meters) that can be walked in 6 min. All participants have to be familiarized with the treadmill exercise protocol. Before the task participants are instructed to set their own pace, to ‘walk as far as you can in 6 minutes’ and to increase or decrease the speed of the treadmill voluntarily. During the task standardized phrases of encouragement are given. The examiner has to monitor the whole task because the participant must maintain a walking pace, defined as at least one foot being weight-bearing at all times. This test has shown to be reliable [44]. Before and after the task,heart rate, oxygen saturation, and Borg Fatigue Scale are controlled.

The International Physical Activity Questionnaire-Short Form (IPAQ-SF) [45]: This tool is used for cross-national monitoring of physical activity and inactivity. This self-administration version asks about three specific types of activity (walking, moderate-intensity activities, and vigorous-intensity activities) over the last 7 days.

### Fatigue

The Piper Fatigue Scale (PFS): The PFS is a validated tool to assess cancer-related fatigue. The PFS-revised (R-PFS) contains 22 items whose scores range from 0 to 10 and includes four dimensions of subjective fatigue: behavioral/severity, affective meaning, sensory, and cognitive/mood. The total fatigue score is calculated. The scale has high reliability (Cronbach’s α=0.96) [46].

The Borg Fatigue Scale [47]: The questionnaire presented a series of numbers arranged vertically from 6 (no perceived exertion) to 20 (maximum perceived exertion) as intensity of perceived exertion. It will be administered before and after carrying out the 6-min walk test.

### Anxiety and depression

The Hospital Anxiety and Depression Scale (HADS) [48,49]: This self-reporting tool assesses the possible presence of anxiety and depression in the setting of a medical non-psychiatric outpatient clinic. It contains 14 items (seven items for each subscale) with four-point Likert scale (ranging from 0 to 3). The global score ranges from 0 to 21 for anxiety and depression. The cutoff point for considering a pathological condition is 11 or above for both subscales.

### Cognitive function

The Trail Making Test (TMT) is one of the most important neuropsychological tests and provides information on speed for attention, sequencing, mental flexibility, visual search, and motor function [50]. The TMT consists of two parts. Part A requires the participant to draw lines to connect consecutive numbers (1 to 25) distributed on a sheet of paper as fast as possible. In part B, the participant must draw a consecutive line between numbers and letters (for example, 1-A-2-B-3-C, and so on). Before each part, a practical example is administered to ensure participants understand each part. When a mistake is made during the test, the examiner corrects it. Scoring is based on the length of time required to finish each part.

The Auditory Consonant Trigram (ACT) is also known as the Brown-Peterson procedure. This tool is used to test short-term memory, attention span, and information-processing capacity in adults [50]. The subject listens to the consonant trigram (CCC) at a rate of one letter per second following by a mental test as counting backward during delay intervals of 9, 18, and 36 s randomly. For the first five consonant trigrams, there is no delay interval (0 seconds) to recall the letters. Afterwards, the subject is asked to recall the trigram. Scoring is based on the total number of letters properly recalled in each delay interval.

### Accelerometry

The protocol for using and analysis of accelerometers has been published previously [51]. Participants will be asked to wear a tri-axial accelerometer (ActiGraph GT3X+, Pensacola, FL, US) over 8 consecutive days, starting the same day they receive the device. They will return the accelerometers to the researcher 9 days later. We will show them how to wear the accelerometer on their lower back with elastic belt all day (including sleeping hours). It is compulsory, for reasons of security, to take off accelerometers during aquatic activities (for example, bathing).

The first day of recording will not be included in the analysis, so a total of 7 days of recording (minimum of 10 h or more of registration per day) will be necessary to be included in the analysis. It will exclude from the analyses bouts of 60 continuous minutes of 0 activity intensity counts, considering these periods as non-wearing time. Monitor wearing time will be calculated by subtracting the non-wear time and the sleeping time (recorded with a diary of days/hours) from the total registered time for the entire day (typically 1,440 min). A recording of >20,000 counts per min will be considered as a potential malfunction of the accelerometer and the value will be excluded from the analyses.

### Lymphedema

Changes in size or volume of the upper limbs are measured to diagnose lymphedema. An inextensible flexible tape 0.5 cm wide × 2 m long with an accuracy of 0.1 cm will be used. According to research [52], the patient must be in an upright sitting position with both arms on a table, shoulders in neutral rotation and flexion of 45°, and forearms at maximum supination, the examiner will have to measure the circumference at 5-cm intervals along both arms, using the elbow fold as the reference beginning point. This procedure has been shown to be valid and reliable [53-55].

### Anthropometric perimeters

Perimeters of waist and hip: We will measure perimeters of waist and hip following the specific method [56]. An inextensible flexible tape 0.5 cm wide x 2 m long with an accuracy of 0.1 cm will be used. The examiner will measure the patient’s waist in a standing position placing the tape measure at the midpoint between the last rib and the upper anterior iliac spine. The measure will take place at the end of exhalation.

For the hip perimeter the patient will remain in a standing position and the examiner will place the tape measure at the midpoint between both trochanters to the level of maximum relief of the buttocks and symphysis pubis.

### Telerehabilitation group/intervention group

The intervention will be implemented by research group called CUIDATE. The e-CUIDATE system is an 8-week program (three training sessions per week) which aims to recover a healthy lifestyle in breast cancer survivors. Participants will have access to a range of exercises such as breathing exercises, mobility, strength, and stretching. All of these exercises will be assigned to women in the telerehabilitation group according to their perceived needs at baseline assessment. These needs will be established based on fatigue level, functional capacity (6-min walk test), and neck-shoulder pain reported by the patients. For this reason each participant will receive progressive personal training (for example, number and type of exercises, repetitions, series, and so on). We will instruct participants to use the telerehabilitation system on day 2 testing (username and password are provided for each participant). The participants’ aims will be set weekly; the CUIDATE group will then choose exercises aimed at achieving these goals. Furthermore women will receive telephone calls, text messages, and videoconference sessions (as required) to resolve any question or suggestion. Efforts will be made to prevent the telerehabilitation group receiving additional physical care.

### Telerehabilitation system

The CUIDATE group will identify impairments of participants at baseline assessment. The aims based on weekly improvements will be set to facilitate the final goal. After each training session there will be feedback between the participant and the CUIDATE group to change or improve any exercise or activity. This feedback will allow to us to select adequate exercises and levels of difficulty. As a result, a modifiable personal training will be assigned to each one focusing on cardio-respiratory, mobility, and endurance performance through online system rehabilitation (using written instructions, HD videos, and audio files).

### Checking through e-CUIDATE

Participants will have three sessions per week during the 8-week period through e-CUIDATE. We will check that assigned sessions are being done properly each week through a control platform. Also, comments about doubts and suggestions will be written by participants after performing their sessions through e-CUIDATE. They will receive feedback (through text messages in their profiles in e-CUIDATE) about these comments before attending the following session. If they have a webcam they will also be able to receive feedback via this videoconferencing facility.

### Telephone calls

The telephone calls will be made by the CUIDATE group. The aim of these calls will be to solve any problems of carrying out the training sessions and to check levels of satisfaction and improvement. Also, messages of encouragement will be given to stimulate adherence to the program.

### Instant messaging through e-CUIDATE

Changes about assigned exercises will be provided by us. More messages of support and encouragement will also be received via this medium. Participants will use instant messaging to advise of any change in training days.

### Videoconference facility through e-CUIDATE

The videoconference facility will be available three times per week with a set schedule through Wormhole Web Conference and Skype software (Microsoft corporation, Redmond, Washington).

A single point of contact between patients and therapists will be located in university facilities and staffed by two full-time researchers. This will ensure that all patients receive standardized and comparable feedback and advice.

### Follow-up at 8 weeks

Post-intervention assessment will be made to check on aims achievement. Support and encouragement will be given to follow with training sessions self-sufficiently because all participants will continue to have access to their profiles in e-CUIDATE.

### Control group/usual care

The control group will receive a dossier of written information with brief general recommendations about stress management and improving physical fitness. These recommendations will be handed in at the beginning of the study. The dossiers will contain similar information to those of the telerehabilitation group but with a single written support. After completion of this study, for ethical compromise of the CUIDATE group, control participants will be given the opportunity to participate in a telerehabilitation program. These data will be not used in this research project.

### Monitoring with accelerometers

Participants of both groups (telerehabilitation and control group) will wear accelerometers (tri-axial motion sensor) at baseline, 8 weeks, and 24 weeks to know the lifestyle and habits of breast cancer survivors at different stages of the study.

### Sample size

**Figure 1:**
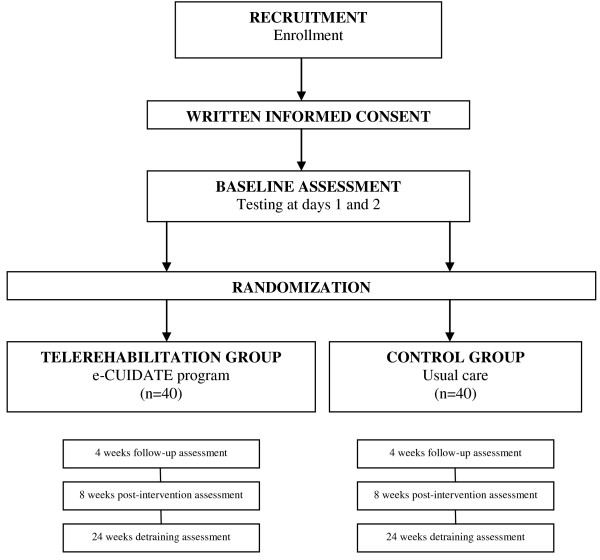
Flow diagram showing the recruitment of patients.

The estimated sample size was determined for the primary outcome variable, that is, overall HRQoL using EORTC QLQ-C30 version 3.0 [26]. According to previously reported data [57] a minimally important difference of this HRQoL instrument is 5 to 10 points. Assuming that telerehabilitation increase HRQoL in breast cancer survivors respect control group we can detect differences of at least 5% with a power of 90% and an α of 0.05 with two groups (telerehabilitation and usual care group) of 36 participants assuming similar standard deviation of approximately 7 points. We will assume a maximum loss at follow-up of 10% [58]. We will recruit a total of 80 breast cancer survivors (that is, one intervention group and one usual care group of 40 persons each). Figure 1 shows the flow diagram of the study participants.

### Randomization and blinding

We allocated patients to a telerehabilitation program or control group in five randomization cycles, using computer-generated numbers (EPIDAT 3.1, Xunta de Galicia). The sequence was entered into numbered opaque envelopes by an external member and they were opened after completion of the baseline assessment. For ethical implications, those participants allocated to the control group, who finished the period of 6 months for the current study, were invited to receive the intervention when last outcome measurement was carried out. The researcher in charge of randomly assigning participants will not know in advance what treatment the next participant would receive and will not participate in assessment. Assessment staff will be blinded to the participant randomization assignment. No changes in assignment will be possible from the staff involved in the telerehabilitation program.

### Data analysis

Mean and standard deviation will be used to represent the variable scores at baseline and different follow-up measurements. Study population will be characterized using different descriptive statistics parameters. In a first step, we will analyze possible differences between groups at baseline using a one-way analysis of variance for continuous data (or equivalent statistical approach in the case of non-parametrical data) and Chi-square for categorical data. For the main analysis we will use an analysis of covariance (ANCOVA) to assess the effects of intervention on study variables. Time since diagnosis, age, tumor stage, and type of oncology surgery treatment will be used as covariates. To complete the analysis we will report effect size and level significance attending to interaction effects (group × time).

### Implications/discussion

The e-CUIDATE telehealth system study will investigate the effects of 8-week of an innovative intervention based on planned physical activity and therapeutic exercise on QoL and previously presented variables. The physical activity purposes include aerobic exercise which has showed to be effective in reducing many major health problems in cancer survivors such as fatigue [59], mobility and strengthening exercises focused on the shoulder area that are necessary for this population to help with pain and disability associated with arm morbidity in this patient group [17,18] and recovery strategies focused in respiratory exercise, relaxation techniques, and flexibility exercises which have been well tolerated in this population. These recovery strategies related to physical training could produce an additional improvement in the patients increasing the adherence to exercise programs in cancer survivors [60]. To our knowledge, this is the first randomized controlled trial specifically designed to assess a telerehabilitation system in breast cancer survivors. QoL improvement is considered an indicator of rehabilitation cancer success [61]. Therefore the results of this study could give support for the use of this type of strategy in an increasing group of 17.8 million prevalent cases of cancer in the European Union [62] with a high proportion of them claiming adequate rehabilitation services. Cancer survivors suffer a chronic illness with most prevalent symptoms during the aging process. National health systems need to benefit from advances in technology to give adequate support to this increasing population. For this reason, the advantages of telehealth systems need to be explored in this setting.

Telehealth systems have shown feasibility and cost-effectiveness in several major health problems [63,64]. The explosive worldwide increase of cancer survivors could generate economic and social costs for national health systems. The tuning process of this type of intervention could benefit the health of the prevalent cancer patients and reduce the costs of this relatively new health problem.

## Trial status

Active recruitment.

## Competing interests

The authors declare that they have no competing interests.

## Authors’ contributions

MAM conceived the study, designed e-CUIDATE, and drafted the manuscript. LDR, ICV, CFL, and NGC participated in the study design and planned the statistical analysis of data. ICV, CFL, AAG, and NGC designed the diary sessions of e-CUIDATE intervention. AAG, LDR, and CFL give considerable facility to relation between hospital centers and university laboratories. RDMA, MLA, and CSS advised on the medical aspect of the protocol and participated in the enrollment of the patients to the study. All authors read and approved the final manuscript.

## Authors’ information

NGC is a lecturer at University of Granada and this project represents her PhD thesis topic. Her particular interest has been the give support to breast cancer during rehabilitation phase. CFL and AAG are physiotherapists and academics, working and researching the area of oncology rehabilitation. ICV is physical exercise specialists and academic researching in the area of exercise in oncology patients. MAM is a sports physician and physiotherapist working as research leader in this project and supervisor of PhD thesis. LDR and CSS are nurses and academic involves in research with cancer survivors. MLA and RDMA are consultant physicians with significant interest in cancer rehabilitation.

## References

[B1] Chen X Lu W Zheng W Gu K Matthews CE Chen Z Zheng Y Shu XO Exercise after diagnosis of breast cancer in association with survival Cancer Prev Res (Phila) 2011 4 1409 1418 10.1158/1940-6207.CAPR-10-0355 21795422 PMC3169008

[B2] Hayes S Rye S Battistutta D Yates P Pyke C Bashford J Eakin E Design and implementation of the Exercise for Health trial - a pragmatic exercise intervention for women with breast cancer Contemp Clin Trials 2011 32 577 585 10.1016/j.cct.2011.03.015 21463707

[B3] Smith SL Singh-Carlson S Downie L Payeur N Wai ES Survivors of breast cancer: patient perspectives on survivorship care planning J Cancer Surviv 2011 5 337 344 10.1007/s11764-011-0185-7 21643836

[B4] Knols RH de Bruin ED Shirato K Uebelhart D Aaronson NK Physical activity interventions to improve daily walking activity in cancer survivors BMC Cancer 2010 10 406 10.1186/1471-2407-10-406 20684789 PMC2921399

[B5] Schmitz KH Balancing lymphedema risk: exercise versus deconditioning for breast cancer survivors Exerc Sport Sci Rev 2010 38 17 24 10.1097/JES.0b013e3181c5cd5a 20016295 PMC2800982

[B6] Kim SH Son BH Hwang SY Han W Yang JH Lee S Yun YH Fatigue and depression in disease-free breast cancer survivors: prevalence, correlates, and association with quality of life J Pain Symptom Manage 2008 35 644 655 10.1016/j.jpainsymman.2007.08.012 18358687

[B7] Cantarero-Villanueva I Fernández-Lao C Fernández-de-las-Peñas C Díaz-Rodríguez L Sánchez-Cantalejo E Arroyo-Morales M Associations among musculoskeletal impairments, depression, body image and fatigue in breast cancer survivors within the first year after treatment Eur J Cancer Care (Engl) 2011 20 632 639 10.1111/j.1365-2354.2011.01245.x 21410803

[B8] Campbell KL Neil SE Winters-Stone KM Review of exercise studies in breast cancer survivors: attention to principles of exercise training Br J Sports Med 2012 46 909 916 10.1136/bjsports-2010-082719 23007178

[B9] Lauridsen MC Overgaard M Overgaard J Hessov IB Cristiansen P Shoulder disability and late symptoms following surgery for early breast cancer Acta Oncol 2008 47 569 575 10.1080/02841860801986627 18465324

[B10] Fernández-Lao C Cantarero-Villanueva I Fernández-de-las-Peñas C Del-Moral-Ávila R Arendt-Nielsen L Arroyo-Morales M Myofascial trigger points in neck and shoulder muscles and widespread pressure pain hypersensitivity in patients with postmastectomy pain: evidence of peripheral and central sensitization Clin J Pain 2010 26 798 806 10.1097/AJP.0b013e3181f18c36 20842013

[B11] Lelieveld OT Armbrust W Geertzen JH de Graaf I van Leeuwen MA Sauer PJ van Weert E Bouma J Promoting physical activity in children with juvenile idiopathic arthritis through an internet-based program: results of a pilot randomized controlled trial Arthritis Care Res (Hoboken) 2010 62 697 703 10.1002/acr.20085 20191468

[B12] Carmichael AR Daley AJ Rea DW Bowden SJ Physical activity and breast cancer outcome: a brief review of evidence, current practice and future direction Eur J Surg Oncol 2010 36 1139 1148 10.1016/j.ejso.2010.09.011 20947287

[B13] Duijts SF Faber MM Oldenburg HS van Beurden M Aaronson NK Effectiveness of behavioral techniques and physical exercise on psychosocial functioning and health-related quality of life in breast cancer patients and survivors-a meta-analysis Psychooncology 2011 20 115 126 10.1002/pon.1728 20336645

[B14] Harrison SA Hayes SC Newman B Age-related differences in exercise and quality of life among breast cancer survivors Med Sci Sports Exerc 2010 42 67 74 20010128 10.1249/MSS.0b013e3181b0f2cb

[B15] Chan DN Lui LY So WK Effectiveness of exercise programmes on shoulder mobility and lymphoedema after axillary lymph node dissection for breast cancer: systematic review J Adv Nurs 2010 66 1902 1914 20626480 10.1111/j.1365-2648.2010.05374.x

[B16] Hack TF Kwan WB Thomas-Maclean RL Towers A Miedema B Tilley A Chateau D Predictors of arm morbidity following breast cancer surgery Psychooncology 2010 19 1205 1212 10.1002/pon.1685 20099254

[B17] Nesvold IL Fosså SD Holm I Naume B Dahl AA Arm/shoulder problems in breast cancer survivors are associated with reduced health and poorer physical quality of life Acta Oncol 2010 49 347 353 10.3109/02841860903302905 19842790

[B18] McNeely ML Campbell K Ospina M Rowe BH Dabbs K Klassen TP Mackey J Courneya K Exercise interventions for upper-limb dysfunction due to breast cancer treatment Cochrane Database Syst Rev 2010 6 CD005211 10.1002/14651858.CD005211.pub2 PMC12861582 20556760

[B19] Loprinzi PD Cardinal BJ Effects of physical activity on common side effects of breast cancer treatment Breast Cancer 2012 19 4 10 10.1007/s12282-011-0292-3 21725654

[B20] Haines TP Sinnamon P Wetzig NG Lehman M Walpole E Pratt T Smith A Multimodal exercise improves quality of life of women being treated for breast cancer, but at what cost? Randomized trial with economic evaluation Breast Cancer Res Treat 2010 124 163 175 10.1007/s10549-010-1126-2 20734132

[B21] McCue M Fairman A Pramuka M Enhancing quality of life through telerehabilitation Phys Med Rehabil Clin N Am 2010 21 195 205 10.1016/j.pmr.2009.07.005 19951786

[B22] Kairy D Lehoux P Vincent C Visintin M A systematic review of clinical outcomes, clinical process, healthcare utilization and costs associated with telerehabilitation Disabil Rehabil 2009 31 427 447 10.1080/09638280802062553 18720118

[B23] Dlugonski D Motl RW McAuley E Increasing physical activity in multiple sclerosis: replicating Internet intervention effects using objective and self-report outcomes J Rehabil Res Dev 2011 48 1129 1136 10.1682/JRRD.2010.09.0192 22234717

[B24] Gautam AP Maiya AG Vidyasagar MS Effect of home-based exercise program on lymphedema and quality of life in female postmastectomy patients: pre-post intervention study J Rehabil Res Dev 2011 48 1261 1268 10.1682/JRRD.2010.05.0089 22234669

[B25] Davies NJ Batehup L Thomas R The role of diet and physical activity in breast, colorectal, and prostate cancer survivorship: a review of the literature Br J Cancer 2011 Suppl 1 S52 S73 22048034 10.1038/bjc.2011.423 PMC3251953

[B26] Aaronson NK Ahmedzai S Bergman B Bullinger M Cull A Duez NJ Filiberti A Flechtner H Fleishman SB de Haes JCJM Kaasa S Klee MC Osoba D Razavi D Rofe PB Schraub S Sneeuw KCA Sullivan M Takeda F The European Organisation for Research and Treatment of Cancer QLQ-C30: A quality-of-life instrument for use in international clinical trials in oncology J Natl Cancer Inst 1993 85 365 376 10.1093/jnci/85.5.365 8433390

[B27] Fayers PM Aaronson NK Bjordal K Groenvold M Curran D Bottomley A The EORTC QLQ-C30 Scoring Manual 2001 3 Brussels: European Organisation for Research and Treatment of Cancer

[B28] Sprangers MA Groenvold M Arraras JI Franklin J te Velde A Muller M Franzini L Williams A de Haes HC Hopwood P Cull A Aaronson NK The European Organization for Research and Treatment of Cancer breast cancer-specific quality-of-life questionnaire module: first results from a three-country field study J Clin Oncol 1996 14 2756 2768 8874337 10.1200/JCO.1996.14.10.2756

[B29] Vanderweeën L Oostendorp RA Vaes P Duquet W Pressure algometry in manual therapy Man Ther 1996 1 258 265 10.1054/math.1996.0276 11440515

[B30] Fernández-Lao C Cantarero-Villanueva I Fernández-de-las-Peñas C Del-Moral-Ávila R Menjón-Beltrán S Arroyo-Morales M Widespread mechanical pain hypersensitivity as a sign of central sensitization after breast cancer surgery: comparison between mastectomy and lumpectomy Pain Med 2011 12 72 78 10.1111/j.1526-4637.2010.01027.x 21143767

[B31] Chesterton LS Sim J Wright CC Foster NE Interrater reliability of algometry in measuring pressure pain thresholds in healthy humans, using multiple raters Clin J Pain 2007 23 760 766 10.1097/AJP.0b013e318154b6ae 18075402

[B32] Jones DH Kilgour RD Comtois AS Test-retest reliability of pressure pain threshold measurements of the upper limb and torso in young healthy women J Pain 2007 8 650 656 10.1016/j.jpain.2007.04.003 17553750

[B33] Cleeland CS Ryan KM Pain assessment: global use of the Brief Pain Inventory Ann Acad Med Singapore 1994 23 129 138 8080219

[B34] The Brief Pain Inventory. User Guide http://www.mdanderson.org/education-and-research/departments-programs-and-labs/departments-and-divisions/symptom-research/symptom-assessment-tools/BPI_UserGuide.pdf

[B35] Badia X Muriel C Gracia A Núñez-Olarte JM Perulero N Gálvez R Carulla J Cleeland CS Validación española del cuestionario Brief Pain Inventory en pacientes con dolor de causa neoplásica Med Clin (Barc) 2003 120 52 59 12570914 10.1016/s0025-7753(03)73601-x

[B36] McQuade KJ Turner JA Buchner DM Physical fitness and chronic low back pain. An analysis of the relationships among fitness, functional limitations, and depression Clin Orthop Relat Res 1988 233 198 204 2969788

[B37] Ruiz-Ruiz J Mesa JL Gutiérrez A Castillo MJ Hand size influences optimal grip span in women but not in men J Hand Surg Am 2002 27 897 901 10.1053/jhsu.2002.34315 12239682

[B38] España-Romero V Ortega FB Vicente-Rodríguez G Artero EG Rey JP Ruiz JR Elbow position affects handgrip strength in adolescents: validity and reliability of Jamar, DynEx, and TKK dynamometers J Strength Cond Res 2010 24 272 277 10.1519/JSC.0b013e3181b296a5 19966590

[B39] Imagama S Matsuyama Y Hasegawa Y Sakai Y Ito Z Ishiguro N Hamajima N Back muscle strength and spinal mobility are predictors of quality of life in middle-aged and elderly males Eur Spine J 2011 20 954 961 10.1007/s00586-010-1606-4 21072545 PMC3099149

[B40] Netz Y Ayalon M Dunsky A Alexander N The multiple-sit-to-stand field test for older adults: what does it measure? Gerontology 2004 50 121 126 10.1159/000076769 15114032

[B41] Ritchie C Trost SG Brown W Armit C Reliability and validity of physical fitness field tests for adults aged 55 to 70 years J Sci Med Sport 2005 8 61 70 10.1016/S1440-2440(05)80025-8 15887902

[B42] Ortega FB Ruiz JR España-Romero V Vicente-Rodriguez G Martínez-Gómez D Manios Y Béghin L Molnar D Widhalm K Moreno LA Sjöström M Castillo MJ The International Fitness Scale (IFIS): usefulness of self-reported fitness in youth Int J Epidemiol 2011 40 701 711 10.1093/ije/dyr039 21441238

[B43] Ortega FB Sánchez-López M Solera-Martínez M Fernández-Sánchez A Sjöström M Martínez-Vizcaino V Self-reported and measured cardiorespiratory fitness similarly predict cardiovascular disease risk in young adults Scand J Med Sci Sportsin press 10.1111/j.1600-0838.2012.01454.x 22417235

[B44] Laskin JJ Bundy S Marron H Moore H Swanson M Blair M Humphrey R Using a treadmill for the 6-minute walk test: reliability and validity J Cardiopulm Rehabil Prev 2007 27 407 410 18197077 10.1097/01.HCR.0000300270.45881.d0

[B45] Craig CL Marshall AL Sjöström M Bauman AE Booth ML Ainsworth BE Pratt M Ekelund U Yngve A Sallis JF Oja P International physical activity questionnaire: 12-country reliability and validity Med Sci Sports Exerc 2003 35 1381 1395 10.1249/01.MSS.0000078924.61453.FB 12900694

[B46] Piper BF Dibble SL Dodd MJ Weiss MC Slaughter RE Paul SM The revised Piper Fatigue Scale: psychometric evaluation in women with breast cancer Oncol Nurs Forum 1998 25 677 684 9599351

[B47] Borg GA Psychophysical bases of perceived exertion Med Sci Sports Exerc 1982 14 377 381 7154893

[B48] Zigmond AS Snaith RP The hospital anxiety and depression scale Acta Psychiatr Scand 1983 67 361 370 10.1111/j.1600-0447.1983.tb09716.x 6880820

[B49] Herrero MJ Blanch J Peri JM De Pablo J Pintor L Bulbena A A validation study of the hospital anxiety and depression scale (HADS) in a Spanish population Gen Hosp Psychiatry 2003 25 277 283 10.1016/S0163-8343(03)00043-4 12850660

[B50] Spreen O Strauss E A Compendium of Neuropsychological Tests: Administration, Norms, and Commentary 1998 New York: Oxford University Press

[B51] Carbonell-Baeza A Ruiz JR Aparicio VA Ortega FB Munquía-Izquierdo D Alvarez-Gallardo IC Segura-Jiménez V Camiletti-Moirón D Romero A Estévez-López F Samos B Casimiro AJ Sierra Á Latorre PA Pulido-Martos M Femia P Pérez-López IJ Chillón P Girela-Rejón MJ Tercedor P Lucía A Delgado-Fernández M Land- and water-based exercise intervention in women with fibromyalgia: the al-Andalus physical activity randomised controlled trial BMC Musculoskelet Disord 2012 13 18 10.1186/1471-2474-13-18 22336292 PMC3350451

[B52] Torres Lacomba M Yuste Sánchez MJ Zapico Goñi A Prieto Merino D Mayoral Del Moral O Cerezo Téllez E Minayo Mogollón E Effectiveness of early physiotherapy to prevent lymphoedema after surgery for breast cancer: randomised, single blinded, clinical trial BMJ 2010 340 b5396 10.1136/bmj.b5396 20068255 PMC2806631

[B53] Karges JR Mark BE Stikeleather SJ Worrell TW Concurrent validity of upper-extremity volume estimates: comparison of calculated volume derived from girth measurements and water displacement volume Phys Ther 2003 83 134 145 12564949

[B54] Taylor R Jayasinghe UW Koelmeyer L Ung O Boyages J Reliability and validity of arm volume measurements for assessment of lymphedema Phys Ther 2006 86 205 214 16445334

[B55] Torres Lacomba M Yuste Sánchez MJ Prieto Merino D Estudio de fiabilidad y reproducibilidad de las medidas cirtométricas en miembros superior e inferior sanos Cuest fisioter 2010 39 166 178

[B56] Franquelo Morales P Serrano Martínez S Moya Martínez P Buendía Bermejo J Sánchez López M Solera Martínez M Notario Pacheco B Asociación entre distintas medidas de Composición Corporal y Factores de Riesgo Cardiovascular en población adulta Rev Clín Med Fam 2008 2 149 15523828048

[B57] Osoba D Rodrigues G Myles J Zee B Pater J Interpreting the significance of changes in health-related quality-of-life scores J Clin Oncol 1998 16 139 144 9440735 10.1200/JCO.1998.16.1.139

[B58] Fernández-Lao C Cantarero-Villanueva I Ariza-García A Courtney CA Fernández-de-las-Peñas C Arroyo-Morales M Water versus land-based multimodal exercise program effects on body composition in breast cancer survivors: a controlled clinical trial Support Care Cancer 2013 21 521 530 10.1007/s00520-012-1549-x 22864470

[B59] Cramp F Byron-Daniel J Exercise for the management of cancer-related fatigue in adults Cochrane Database Syst Rev 2012 11 CD006145 10.1002/14651858.CD006145.pub3 PMC8480137 23152233

[B60] Cantarero-Villanueva I Fernández-Lao C Del Moral-Avila R Fernández-de-Las-Peñas C Feriche-Fernández-Castanys MB Arroyo-Morales M Effectiveness of core stability exercises and recovery myofascial release massage on fatigue in breast cancer survivors: a randomized controlled clinical trial Evid Based Complement Alternat Med 2012 2012 620619 21792370 10.1155/2012/620619 PMC3139905

[B61] Baili P Hoekstra-Weebers J Van Hoof E Bartsch HH Travado L Garami M Di Salvo F Micheli A Veerus P Cancer rehabilitation indicators for Europe Eur J Cancer 2013 49 1356 1364 10.1016/j.ejca.2012.10.028 23237740

[B62] Gatta G van der Zwan JM Casali PG Siesling S Dei Tos AP Kunkler I Otter R Licitra L Mallone S Tavilla A Trama A Capocaccia R Rare cancers are not so rare: the rare cancer burden in Europe Eur J Cancer 2011 47 2493 2511 10.1016/j.ejca.2011.08.008 22033323

[B63] Kesavadev J Shankar A Pillai PB Krishnan G Jothydev S Cost-effective use of telemedicine and self-monitoring of blood glucose via Diabetes Tele Management System (DTMS) to achieve target glycosylated hemoglobin values without serious symptomatic hypoglycemia in 1,000 subjects with type 2 diabetes mellitus--a retrospective stu Diabetes Technol Ther 2012 14 772 776 10.1089/dia.2012.0088 22734662

[B64] Isetta V Lopez-Agustina C Lopez-Bernal E Amat M Vila M Valls C Navajas D Farre R Cost-effectiveness of a new internet-based monitoring tool for neonatal post-discharge home care J Med Internet Res 2013 15 e38 10.2196/jmir.2361 23419609 PMC3636285

